# Successful Radiotherapy for Metastatic Basal Cell Carcinoma to the Parotid Gland in a Patient With Gorlin-Goltz Syndrome

**DOI:** 10.7759/cureus.67152

**Published:** 2024-08-18

**Authors:** Stefan Neaga, Cristina Beiu, Liliana G Popa, Cristina M Orlov Slavu, Andrei W Anghel

**Affiliations:** 1 Dermatology, Elias Emergency University Hospital, Bucharest, ROU; 2 Oncologic Dermatology, Elias Emergency University Hospital, Carol Davila University of Medicine and Pharmacy, Bucharest, ROU; 3 Oncology, Elias Emergency University Hospital, Bucharest, ROU; 4 Radiotherapy, Elias Emergency University Hospital, Bucharest, ROU

**Keywords:** basal cell carcinoma, metastatic, radiotherapy, nevoid basal cell carcinoma syndrome, gorlin-goltz syndrome

## Abstract

Gorlin-Goltz syndrome (GGS), also known as nevoid basal cell carcinoma syndrome (NBCCS), is an autosomal dominant condition characterized by a predisposition to multiple basal cell carcinomas (BCCs) and other neoplasms and is commonly associated with pathogenic variants in the PTCH1 or SUFU tumor suppressor genes. However, the absence of these genetic markers does not preclude the diagnosis due to the variable genetic expression of the syndrome. Diagnosis relies on a set of established major and minor criteria, particularly when genetic testing fails to identify the typical pathogenic variants.

The primary clinical manifestation of GGS is the development of multiple BCCs. While these typically exhibit slow growth and remain localized, they can manifest more aggressive behavior in individuals with GGS, including local invasiveness and metastatic potential. Moreover, patients with GGS display heightened sensitivity to ionizing radiation, leading to general contraindications for radiation therapy (RT) due to the risk of inducing additional BCCs.

Despite these concerns, we report a case where RT was the only feasible treatment for an inoperable BCC that had metastasized to the parotid gland in a GGS patient. The successful use of RT, which resulted in a cure without adverse effects, illustrates that RT may be a viable option for some GGS patients, reflecting individual variability in radiation sensitivity. This case underscores the importance of personalized treatment plans in managing the complex presentations of GGS, challenging the traditional constraints regarding the use of RT in these patients and suggesting the potential for its reconsideration under specific circumstances.

## Introduction

Gorlin-Goltz syndrome (GGS), also recognized as nevoid basal cell carcinoma syndrome (NBCCS), is an autosomal dominant, multisystem disorder characterized by a high predisposition to basal cell carcinomas (BCCs) and other neoplasms. This condition primarily results from pathogenic variants in the PTCH1 or SUFU tumor suppressor genes [1]. While the presence of these variants confirms the diagnosis, their absence does not necessarily rule it out due to the syndrome's variable genetic expression [2].

The predominant clinical feature of GGS is the emergence of multiple BCCs. These carcinomas can occur alongside a spectrum of other benign and malignant neoplasms. The syndrome is diagnosed based on a set of major and minor criteria [3], which become particularly crucial for establishing a diagnosis in cases where genetic testing does not reveal the pathogenic variants typically associated with GGS [2].

While BCCs typically exhibit slow growth and remain localized, their behavior in individuals with GGS can be more aggressive [4]. Although metastasis of BCCs is rare, when it does occur, it predominantly affects the lymph nodes, lungs, and bones. Notably, approximately 85% of metastatic BCC cases in GGS patients occur within the head and neck area [5].

Additionally, individuals with this genetic syndrome exhibit heightened sensitivity to ionizing radiation. Unlike the approach to sporadic BCCs, radiation therapy (RT) is typically contraindicated for patients with GGS due to the consequent risk of inducing new BCCs [6].

However, here we report a case in which RT was the sole viable treatment option for an inoperable BCC that had metastasized to the parotid gland in a patient with GGS. The successful application of RT achieved a cure without adverse effects, highlighting the importance of personalized treatment plans in managing this complex syndrome.

## Case presentation

A 55-year-old Caucasian male was referred to our Dermatology Department in October 2023 for ongoing dermatological surveillance following oncological intervention for BCC metastasis to the parotid gland. The patient had a notable history of multiple BCCs, each addressed with surgical excision. He also exhibited numerous risk factors associated with BCC, including a history of chronic sun exposure, sunburns, as well as Fitzpatrick skin type I.

Starting in June 2021, in another medical center, the patient underwent several cutaneous surgeries to remove multiple ulcerated, infiltrative, nodular BCCs located at various anatomical sites, including the thorax, left frontal, right frontal, left temporal, and right infraorbital regions. In the majority of these cases, the surgeries achieved clear margins (Table 1).

**Table 1:** Patient’s history of multiple BCCs prior to the presentation at our dermatology department. BCCs: basal cell carcinomas

Number	Location of the BCCs	Resection margins
1	Thorax	Negative
2	Left frontal region	Negative
3	Right frontal region	Positive
4	Left frontal region	Negative
5	Left temporal region	Negative
6	Right infraorbital region	Negative

However, the BCC located on the right frontal area presented positive resection margins, necessitating adjunctive treatment with RT, which consisted of multiple courses of electron beam therapy for a total dosage of 58 Gy. The patient tolerated the procedure well and did not present a local relapse of the tumor.

**Figure 1:**
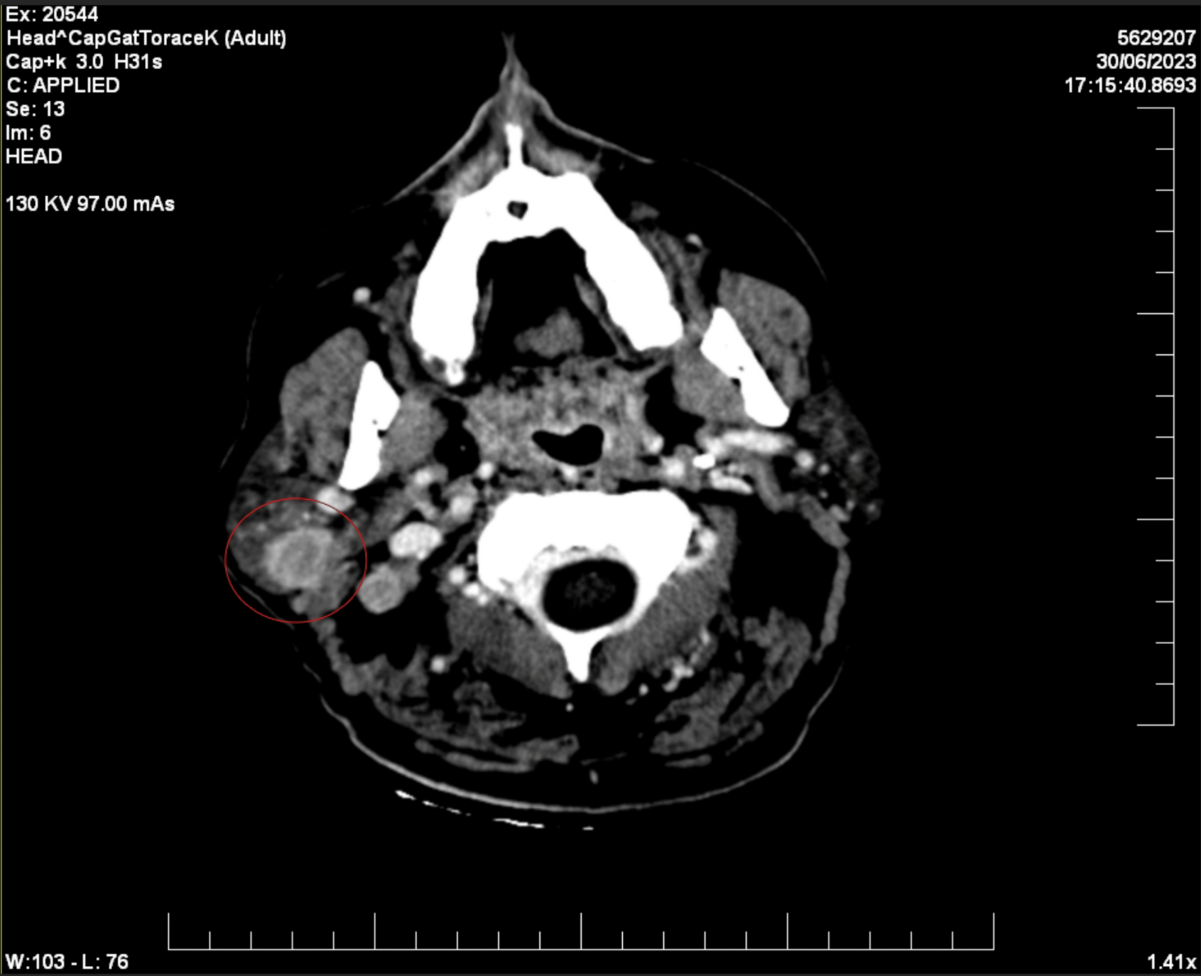
CECT showing a nodular mass within the right parotid gland (red circle). CECT: contrast-enhanced computed tomography

**Figure 2:**
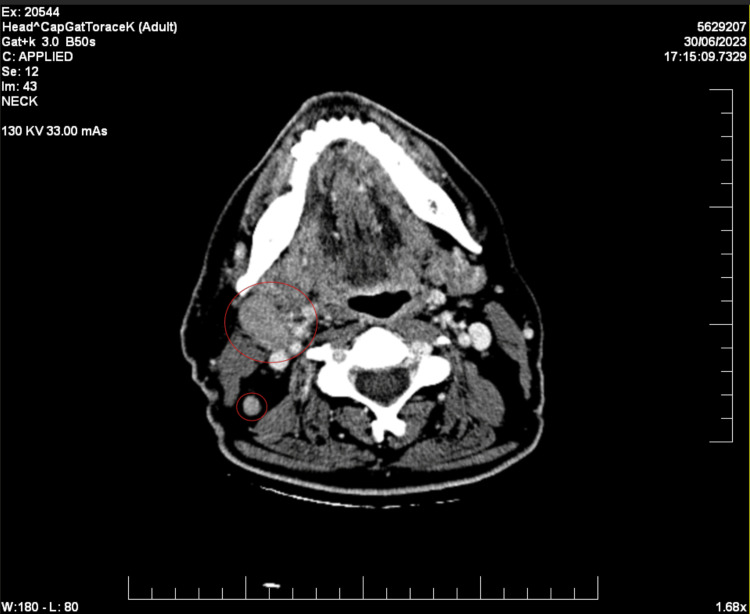
CECT showing lymphadenopathies in the right laterocervical and submandibular regions (red circles). CECT: contrast-enhanced computed tomography

Two years later, in June 2023, a clinically palpable mass was identified inferior to the right auricle. A contrast-enhanced computed tomography (CECT) scan uncovered a nodular mass within the right parotid gland, measuring 2.7×1.6 cm, displaying signs of central necrosis (Figure 1). The scan also revealed lymphadenopathies in the right laterocervical and submandibular regions, in proximity to the submandibular gland and the sternocleidomastoid muscle (Figure 2).

Surgical intervention was undertaken by the plastic surgery team, involving parotidectomy with facial nerve dissection, as well as internal jugular and right jugulodigastric lymphadenectomy using a pre, retro, and subauricular incision. Subsequent histopathological examination confirmed findings consistent with BCC of the parotid gland with prominent perineural invasion and involvement of the right retrodigastric lymph nodes while showing negative results for the right internal jugular lymph nodes. Immunohistochemistry staining was positive for BER-EP4, cytokeratin 7, P63 and negative for SOX10, GATA3, TTF1, CDX2, and CD117. Given the clinical context, the histopathology and immunohistochemical features were interpreted as consistent with basal cell adenocarcinoma of the parotid gland.

In August 2023, a comprehensive imagistic follow-up was undertaken, and the cervical MRI highlighted gadolinium enhancement in the tissue proximal to the post-ablation area, with dimensions of 12x10 mm, alongside the presence of multiple cervical lymphadenopathies. Upon confirmation of incomplete resection of the locally advanced BCC, the case was brought before a multidisciplinary tumor board, comprising specialists from plastic surgery, otolaryngology, radiotherapy, and oncology. The board concluded that re-excision was not a viable option and, consequently, adjuvant RT was determined to be the preferred modality for further management of the patient's condition.

**Figure 3:**
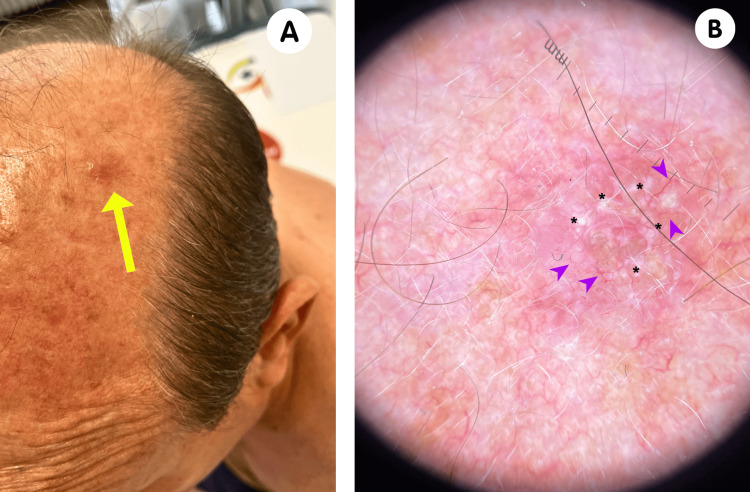
Clinical and dermoscopic aspects of the frontoparietal BCC. Clinical image depicting a pearly, dome-shaped, well-defined, translucent nodular lesion indicated by a yellow arrow (A). Dermoscopic image illustrating branching telangiectases marked with arrowheads and white shiny structures denoted by asterisks (B). BCC: basal cell carcinoma

During his initial visit to our clinic in October 2023, the patient underwent clinical and dermoscopic assessment. Multiple scars from previous BCC excisions were observed. Additionally, a pink nodular lesion with a translucent appearance was noted on the left frontoparietal region of the scalp (Figure 3A). This lesion displayed branching telangiectasias and white shiny structures, which are dermoscopic features characteristic of BCC (Figure 3B).

**Figure 4:**
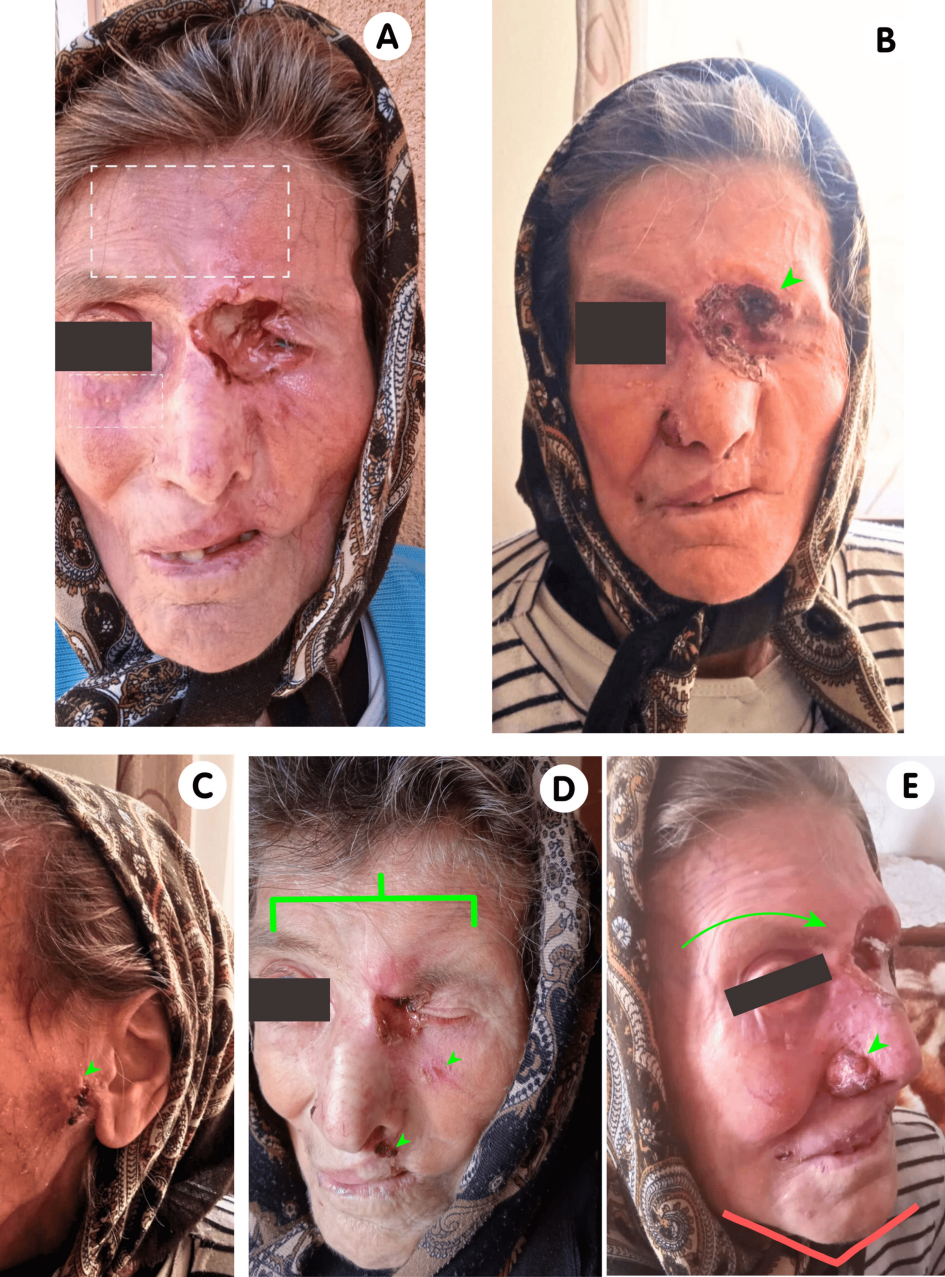
Clinical manifestations in the patient's mother. A range of phenotypic features alongside distinctive dermatological conditions are noticeable as follows: milia scattered on the face among the BCCs (dashed white square) (A); ocular locally destructive terebrant BCC with mutilating evolution (arrowhead) (B); preauricular, nasal, perioral, and zygomatic ulcerated BCCs (arrowheads) (C-E); hypertelorism with widened nasal bridge (green bracket) (D); arched eyebrow (green arrow) and mandibular prognathism (red angle) (E). The authors have obtained the patient's consent to use these images. BCC: basal cell carcinoma

Anamnesis revealed a notable family history; the patient's mother was diagnosed with multiple BCCs, with documented cases affecting the nasal, preauricular, perioral, zygomatic, and ocular regions. Notably, an ocular BCC exhibited a terebrant and mutilating evolution, leading to local destruction. Furthermore, an evaluation of the provided photographic documentation highlighted distinctive facial features in the mother, including macrocephaly, hypertelorism, a widened nasal bridge, arched eyebrows, prognathism of the jawline, and the presence of multiple milia (Figures 4A-4E).

Considering the patient's multiple BCCs and the mother's history of numerous aggressive BCCs alongside suggestive facial traits, a high index of clinical suspicion for GGS was raised. Genetic testing was conducted for mutations in the PTCH1 and SUFU genes. However, the results of these tests were negative.

**Figure 5:**
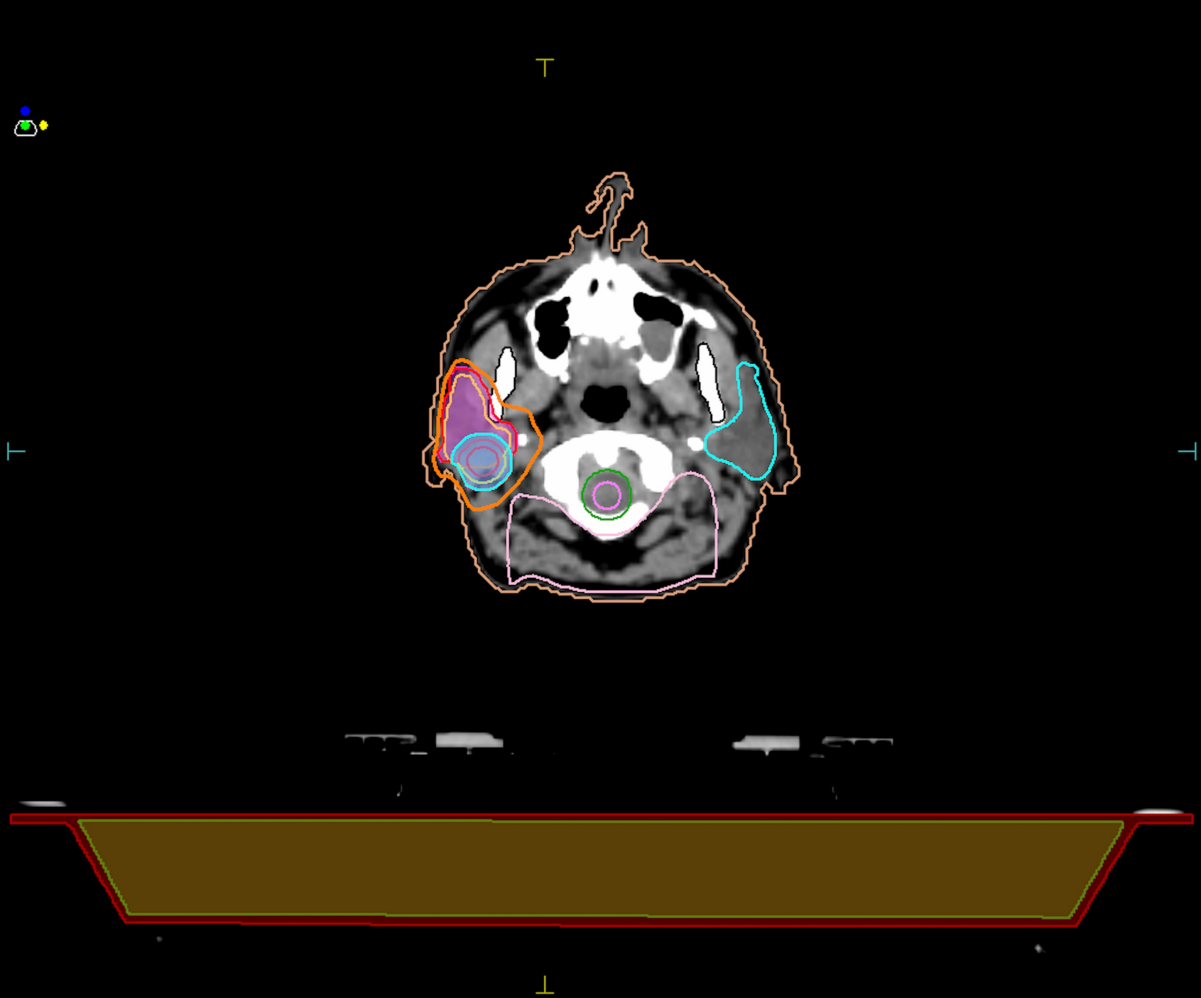
CT-based RT treatment planning showing a transverse (axial) slice at the level of the parotid glands. The RT regimen employed a SIB approach, delivering a total dose of 70 Gy to the right parotid tumor bed in 35 fractions, with a daily dose of 2 Gy per fraction. Concurrently, regions identified with high-risk lymph nodes received 63 Gy, also administered in 35 fractions at 1.8 Gy per fraction, while elective lymph node areas were treated with 56 Gy, delivered in 35 fractions with a daily dose of approximately 1.6 Gy per fraction. CT: computed tomography; RT: radiation therapy; SIB: simultaneous integrated boost

**Figure 6:**
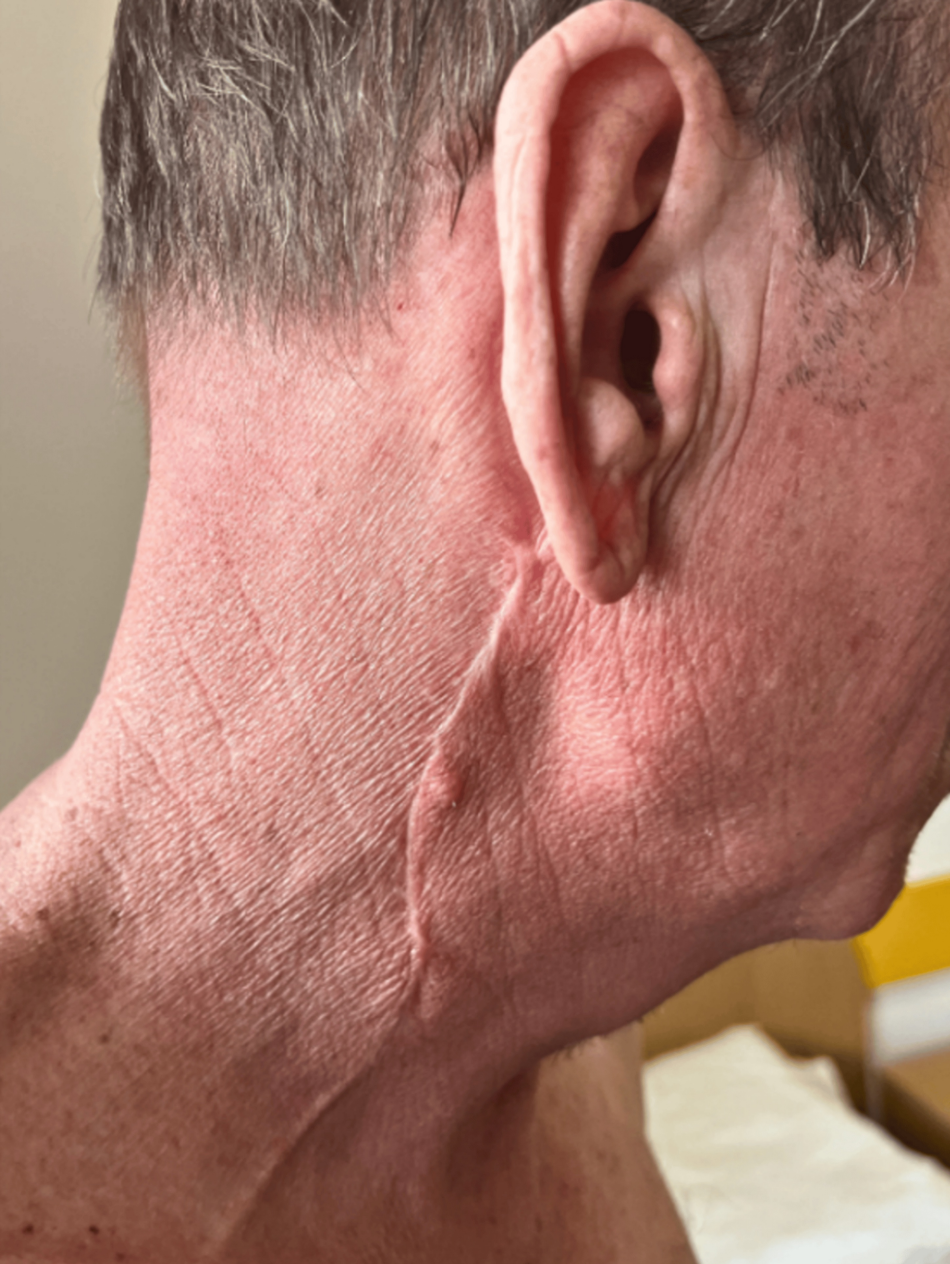
Clinical image of irradiation site three months post-treatment. The irradiated skin and the scar from the parotidectomy show favorable healing with no adverse events noted.

While clinical suspicion for GGS remained elevated, we underscored the necessity of RT as the sole treatment option available for this patient, although approached with significant caution. Starting in December 2023, the patient underwent eight weeks of external beam radiation therapy (RT) utilizing the volumetric modulated arc therapy (VMAT) technique, a form of intensity-modulated radiation therapy (IMRT) that optimizes dose distribution with rotational arc movements. Using a simultaneous integrated boost (SIB) approach, the treatment was administered over 35 fractions, with a total dose of 70 Gy delivered to the right parotid tumor bed (2 Gy per fraction), 63 Gy to areas with high-risk lymph nodes (1.8 Gy per fraction), and 56 Gy to elective lymph node areas (1.6 Gy per fraction) (Figure 5). The patient completed the prescribed course of RT without reporting any associated side effects (Figure 6).

Close dermatological follow-up was advised to promptly detect any local recurrences or new skin malignancies that might arise post-treatment course, with a re-evaluation scheduled every three months. Strict sun protective measures were also employed.

**Figure 7:**
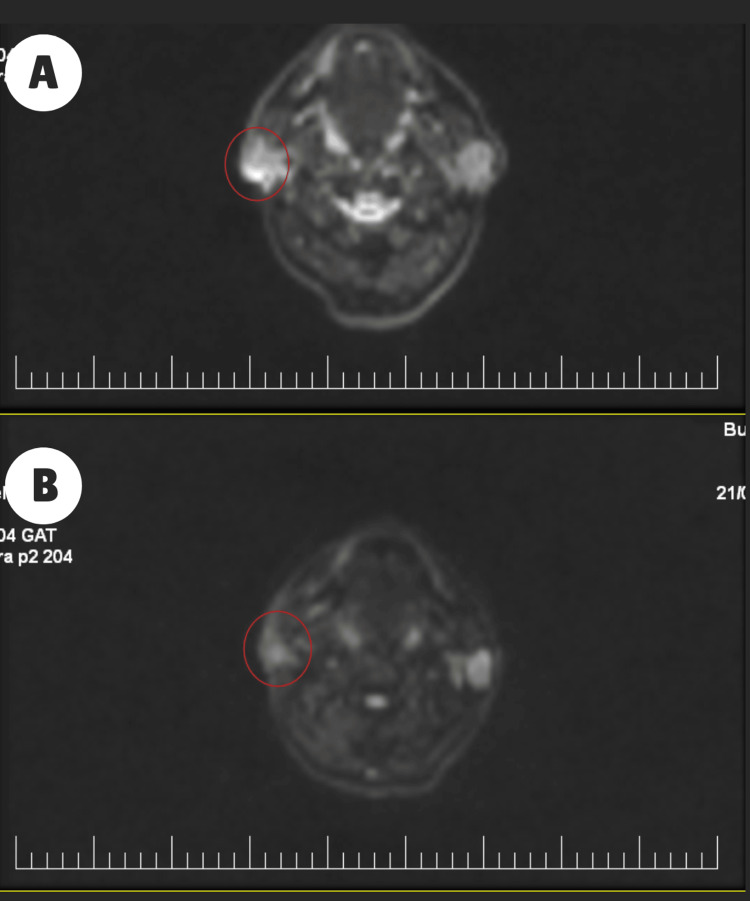
Diffusion-MRI showing the evolution of the parotid mass (red circle) before RT (A) and after RT (B). MRI: magnetic resonance imaging; RT: radiotherapy

**Figure 8:**
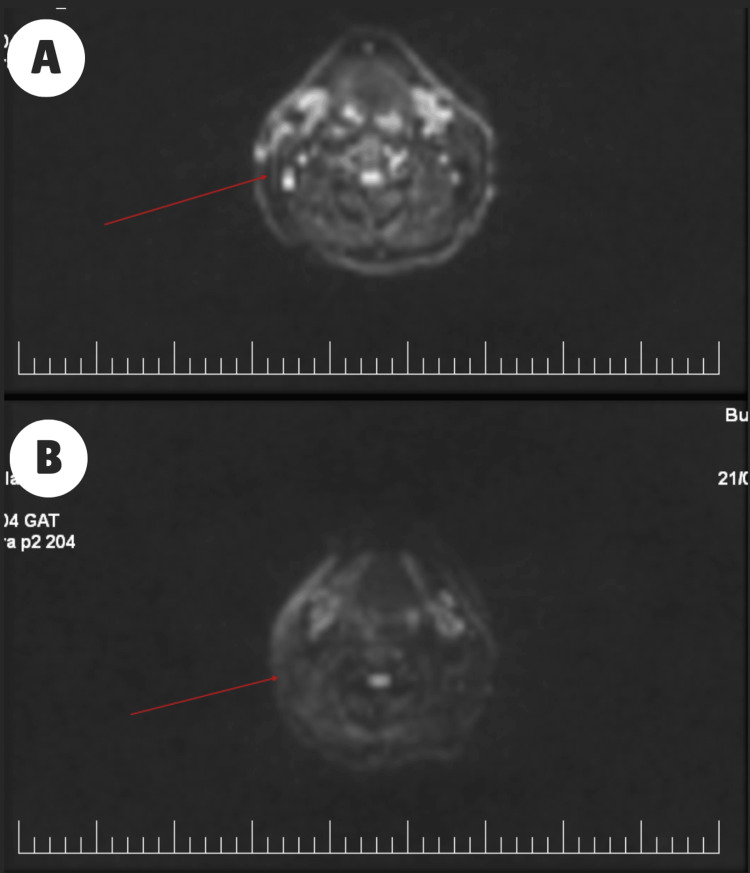
Diffusion MRI showing the evolution of the lymphadenopathies (marked with red arrow) before RT (A) and after RT (B). MRI: magnetic resonance imaging; RT: radiotherapy

Six months after the initial scan, a diffusion-MRI follow-up scan demonstrated a clear positive response to the treatment. The tumoral mass previously observed in the remaining posterior portion of the parotid gland had completely resolved (Figures 7A, 7B). Furthermore, the upper right jugular adenopathy, previously evident, was no longer observable (Figures 8A, 8B).

## Discussion

BCC is the most prevalent skin cancer. The natural history of most BCCs consists of localized, slow growth rate and low metastatic potential [7]. However, certain cases can become locally invasive and destructive or even show metastatic potential. Locoregional or distant metastases are extremely rare phenomena with a documented incidence of only 0.0028-0.5% [8]. However, such events occur in high-risk BCCs and imply high mortality rates, necessitating immediate treatment reassessment [8,9].

Metastatic BCCs most frequently appear in regional lymph nodes, lungs, and bones, particularly seen in cases where lesions are large and invade the cartilage, bone, or skeletal muscle. The head and neck region is the most common primary site of metastasis [5]. Metastasis in the parotid gland is an even rarer phenomenon. The precise incidence of patients with this condition is not very well known, as few cases of metastatic BCC in the parotid region have been described in the literature [10].

Management strategies for patients presenting with advanced BCC encompass surgical intervention and/or RT, particularly for those with incomplete tumor resection or when surgery is not feasible [6]. In cases where metastatic or locally advanced BCC is not suitable for surgical or RT interventions, systemic treatment options are considered, dependent upon availability. The preferred systemic therapy is vismodegib, an inhibitor of the hedgehog signaling pathway. An alternative option involves checkpoint inhibitor immunotherapy, specifically with the programmed cell death 1 (PD-1) inhibitor cemiplimab. Additionally, enrollment in clinical trials offers a viable option where accessible [6].

In our clinical setting, due to the unavailability of both vismodegib and cemiplimab, RT emerged as the sole viable option, particularly as re-excision was deemed infeasible. External beam RT is administered in a fractionated schedule, during consecutive weekdays for several weeks. Despite earlier concerns about the efficacy of RT for advanced BCCs, recent advancements in treatment techniques, notably VMAT, have markedly enhanced patient outcomes, achieving high rates of tumor control and positive cosmetic and functional results [11].

The greatest disadvantage is that RT may induce cutaneous malignancies within irradiated sites, such as basal cell and squamous cell cancers [11]. Furthermore, there's a consensus that RT should be avoided in individuals with GGS, attributed to their pronounced vulnerability to ionizing radiation and the risk of induction of new BCCs [12]. RT may induce aberrant stimulation of DNA synthesis, a phenomenon that can lead to additional mutations, potentially facilitating oncogenesis in the irradiated cells of GGS patients. Consequently, due to the heightened risk of tumor genesis associated with such genomic instability, the administration of RT is generally contraindicated in the management of patients with GGS [12].

Several rare syndromes describe the occurrence of multiple BCCs, with GGS representing the most prevalent among them. Most cases are caused by genetic abnormalities in the PTCH1, PTCH2, or SUFU genes [13]. However, similar to the findings in our patient, there are cases where no pathogenic variant is identified, yet other hallmarks support the diagnosis [2].

Based on the clinical presentation and personal and family medical history, we hypothesized that our patient suffered from GGS. There are more than 100 clinical abnormalities reported in this syndrome, categorized into major and minor features. In the absence of genetic confirmation, a diagnosis of GGS can be confirmed by fulfilling two major criteria or one major criterion alongside two minor criteria [3]. The major criteria underlined in our case are the development of multiple BCCs, which represent the very hallmark of this condition with the presence of a positive family history, as suspected in the patient’s mother.

The presence of the tumors mainly in sun-exposed areas underscores the chronic sun exposure of our patient as a co-factor that may exacerbate the inherent genetic predisposition to tumor formation. BCCs can also become more aggressive in patients diagnosed with GGS, a fact that is highlighted in our case by the presence of metastasis in the parotid gland [4].

Regarding the presence of minor features, we highlight the craniofacial anomalies (macrocephaly, frontal bossing, hypertelorism, high-arched eyebrows, and mandibular prognathism), as well as multiple milia on the face present in the mother.

The management of GGS necessitates a collaborative, multidisciplinary approach due to the scarcity of extensive studies that explore comprehensive treatment modalities aimed at preventing the recurrence of BCCs [6,14]. In our case, the multidisciplinary tumor board decided that RT was the most appropriate treatment course, considering the presence of metastatic BCC that was not fully resected, the impracticality of re-excision, and the unavailability of alternative treatments such as vismodegib and cemiplimab.

Additional considerations included the patient's history of undergoing RT for another BCC without any adverse effects recorded during the two-year follow-up period and the failure of molecular genetic testing to identify pathogenic variants in the PTCH1 or SUFU genes. Nonetheless, we acknowledge that the absence of these genetic markers does not negate the diagnosis of GGS, as evidenced by literature showing only two-thirds of cases test positive for these variants [2]. Furthermore, literature indicates that RT has been successfully employed in managing cases of GGS, despite general reservations due to the increased risk of radiation-induced BCCs in these patients [12].

Considering the above, it is tempting to hypothesize that patients with GGS who do not harbor mutations in the PTCH1 or SUFU genes might be less contraindicated for RT. These factors collectively underscore the complexity of treating GGS and highlight the tailored, patient-centric approach required in its management, particularly when therapeutic options are limited.

The patient underwent RT and remained under strict dermatological surveillance since the risk of developing new BCCs in the irradiated area remains elevated indefinitely [6,11]. Therefore, we strongly consider that a personalized follow-up strategy is imperative, encompassing regular dermatological evaluations, education on self-examination techniques, and guidance on sun protective measures [6,15]. This comprehensive approach aims not only to mitigate the risk of developing new BCCs, especially in areas previously exposed to radiation, but also to ensure the early detection of any BCCs that may develop, thereby optimizing the management and outcome of GGS.

## Conclusions

BCCs, while typically non-metastatic, can exceptionally metastasize a phenomenon that appears to be more prevalent in the context of genetic syndromes such as GGS. This observation underscores the need for further research to elucidate the potential link between GGS and the increased propensity for BCC metastasis. Moreover, the diagnosis of GGS presents unique challenges, particularly in cases where genetic testing fails to identify pathogenic variants. It's crucial for healthcare professionals to recognize that GGS can be diagnosed without detectable genetic mutations, emphasizing the role of clinical observation and patient history in identifying the syndrome.

Despite general recommendations against using RT in GGS due to the risk of inducing further carcinomas, individual variability in radiation sensitivity suggests that RT could be a viable option for some patients. This variability indicates the potential for a personalized approach to treatment, underscoring the importance of further study and clinical vigilance.
